# Acute hemiplegia as initial presentation in FIP1L1-PDGFRA-rearranged myeloid neoplasm with eosinophilia: a case report

**DOI:** 10.3389/fonc.2026.1628690

**Published:** 2026-02-10

**Authors:** Min Cheng, Wenjie Shi, Jin Xu, Doudou Wei, Yue Sun, Qian Jiang, Yongjie Li

**Affiliations:** 1 Department of Neurology, People’s Hospital, Peking University, Beijing, China; 2 Department of Neurology, Jishan County People’s Hospital of Shanxi Province, Shanxi, China; 3 Beijing Key Laboratory of Hematopoietic Stem Cell Transplantation, Peking University People’s Hospital, Peking University Institute of Hematology, National Clinical Research Center for Hematologic Disease, Beijing, China

**Keywords:** case report, eosinophilia, FIP1L1-PDGFRA, imatinib, myeloid tumors

## Abstract

**Background:**

Myeloid neoplasms harboring the FIP1L1-PDGFRA *(F/P)* fusion gene infrequently manifest as acute cerebral infarction. The F/P fusion gene induces proliferation within the eosinophilic lineage, resulting in a clonal hypereosinophilic syndrome that may lead to cerebral infarction. This condition demonstrates a high responsiveness to imatinib therapy.

**Case presentation:**

A 27-year-old man presented with acute hemiplegia and was found to have significant eosinophilia. Neuroimaging revealed acute-subacute infarcts with concurrent focal stenosis of the right middle cerebral artery. He was diagnosed with F/P-rearranged myeloid neoplasm associated with myeloid sarcoma. Treatment with low-dose imatinib monotherapy (100 mg/day) resulted in rapid resolution of both eosinophilia and the vascular stenosis. Over 9 years of follow-up without adjuvant antithrombotic agents, he has maintained sustained molecular remission and normal neurological function, with no recurrent stroke.

**Conclusion:**

This case provides new clinical data for the diagnosis and treatment of this type of eosinophilia, and highlights the importance of early recognition, workup, and treatment. Peripheral hypereosinophilia may cause tissue damage, leading to hypereosinophilic syndrome with cerebral infarction. *F/P*+ clonal hypereosinophilic syndrome is a rare diagnosis to consider in patients with unexplained cerebral infarction and hypereosinophilia. In these instances, it is imperative to conduct a peripheral blood test for the *F/P* fusion gene early in the diagnostic evaluation of hypereosinophilic syndrome. Upon confirmation of the diagnosis, initiation of imatinib therapy should occur promptly. This treatment approach has resulted in a swift and sustained complete cytologic and molecular remission, no recurrence of cerebral infarction, obviating the need for intensive chemotherapy, statins, anticoagulant or anti-platelet agents.

## Introduction

Hypereosinophilia (HE) is a condition where peripheral blood eosinophils exceed 1500 cells/mm³ for over 4 weeks. It can be reactive, neoplastic, or idiopathic. Persistent eosinophil overproduction can lead to organ damage, termed hypereosinophilic syndrome (HES), with an incidence rate of 0.036 per 100, 000 person-years (1). In about 10% of HES patients, the FIP1L1 gene fuses with PDGFRA, forming an active tyrosine kinase that drives myeloid proliferation and can result in FIP1L1-PDGFRA *(F/P)*-positive myeloid neoplasm with eosinophilia (F/P+ MN-eo). This primary cause of clonal HES responds well to imatinib (2). In clinical practice, patients with *F/P*+ MN-eo frequently exhibit eosinophilic infiltration across multiple organs. The diagnosis is primarily dependent on the performance of a bone marrow biopsy and the identification of *F/P* fusion gene expression (3).

In this report, we present the case of a young male patient who experienced an acute hemiplegia cerebral infarction and was subsequently diagnosed with eosinophilia in conjunction with a myeloid tumor characterized by aberrant *F/P* expression. The rearrangement of the PDGFRA gene delineates a specific category of hematopoietic neoplasms, which frequently manifest with persistent eosinophilia and demonstrate high sensitivity to low-dose imatinib mesylate therapy. Administration of imatinib led to a complete and sustained clinical, hematological, and molecular remission within three months of initiating treatment.

## Case information

**Figure 1:**
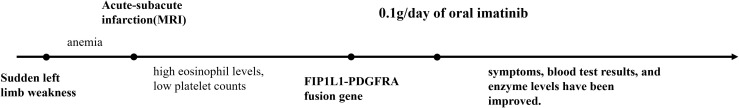
A comprehensive timeline chart detailing the diagnosis and treatment of the case with a series of meticulously organized events, illustrating each significant milestone from the initial presentation of symptoms to the final resolution of the condition.

On admission (Day 0), a 27-year-old man presented with sudden left limb weakness, dizziness, and drooling, diagnosed as acute cerebral infarction. He had no hypertension or diabetes but had allergies. Two weeks earlier, he had mosquito bites, ate raw meat, and had a 10-day cold without fever. Upon admission, the patient presented with anemia, but there was an absence of bleeding or lymphadenopathy. The patient was alert and articulate, exhibiting no cortical dysfunction or cognitive impairment. Neurological examination revealed left-sided facial and central tongue paralysis. Muscle tone was within normal limits, with muscle strength graded as IV+ on the left side and V on the right side. The finger-nose-finger test yielded normal results, and pain sensation in the limbs was symmetrical. Reflexes were within normal limits, and the Babinski sign was potentially positive bilaterally. There was an absence of nuchal rigidity (**Figure 1**).

Various auxiliary tests were conducted. The results of routine blood analysis are presented in **Supplementary Table 1** and **Supplementary Figures 1B, C**. The results of biochemical tests were as follows: creatinine, 132 μmol/L; uric acid, 445 μmol/L; triglycerides, 6.44 mmol/L; lactate dehydrogenase, 419 U/L; and α-hydroxybutyrate dehydrogenase, 370 U/L. Tests for disseminated intravascular coagulation returned the following results: prothrombin time, 13.3 s (normal: 9.40–12.50 s); activated partial thromboplastin time, normal; international normalized ratio, 1.24 (normal: 0.8–1.5); and d-dimer, 752 ng/mL. No abnormalities were detected in viral antibodies, thyroid function, parasitic antibodies, or autoantibodies. No parasitic eggs were detected in stool cultures.

**Figure 2:**
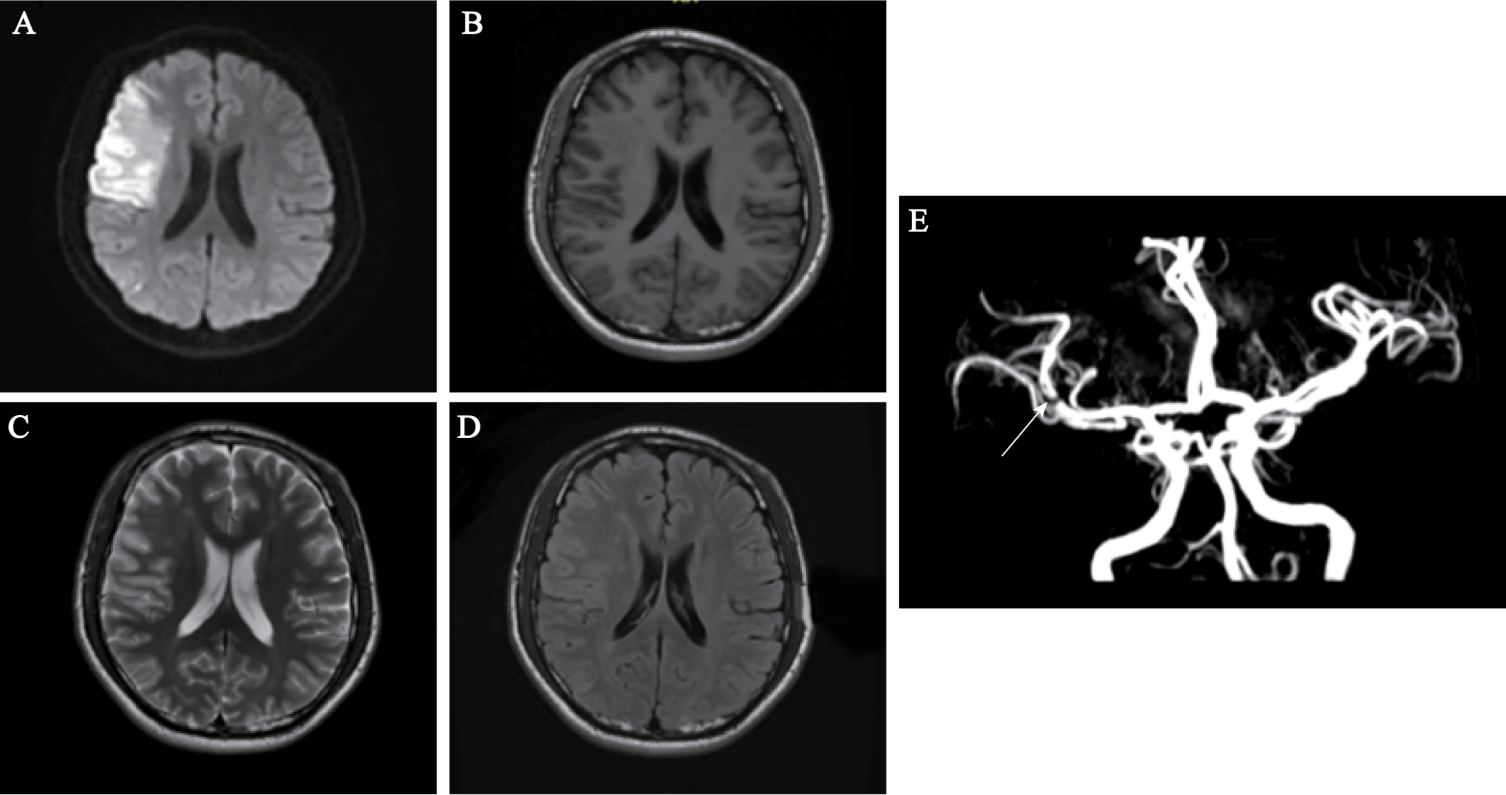
Brain MRI and MRA findings at admission. **(A, B)** Axial diffusion-weighted imaging (DWI) showing hyperintense signals in the left cerebellar hemisphere, indicating acute infarction. **(C, D)** Axial fluid-attenuated inversion recovery (FLAIR) sequences revealing hyperintense lesions in the right frontal and temporal lobes, suggestive of subacute infarction. **(E)** Magnetic resonance angiography (MRA) demonstrating focal stenosis in the right middle cerebral artery (MCA) M1 segment (arrow), with attenuated distal flow signals.

**Figure 3:**
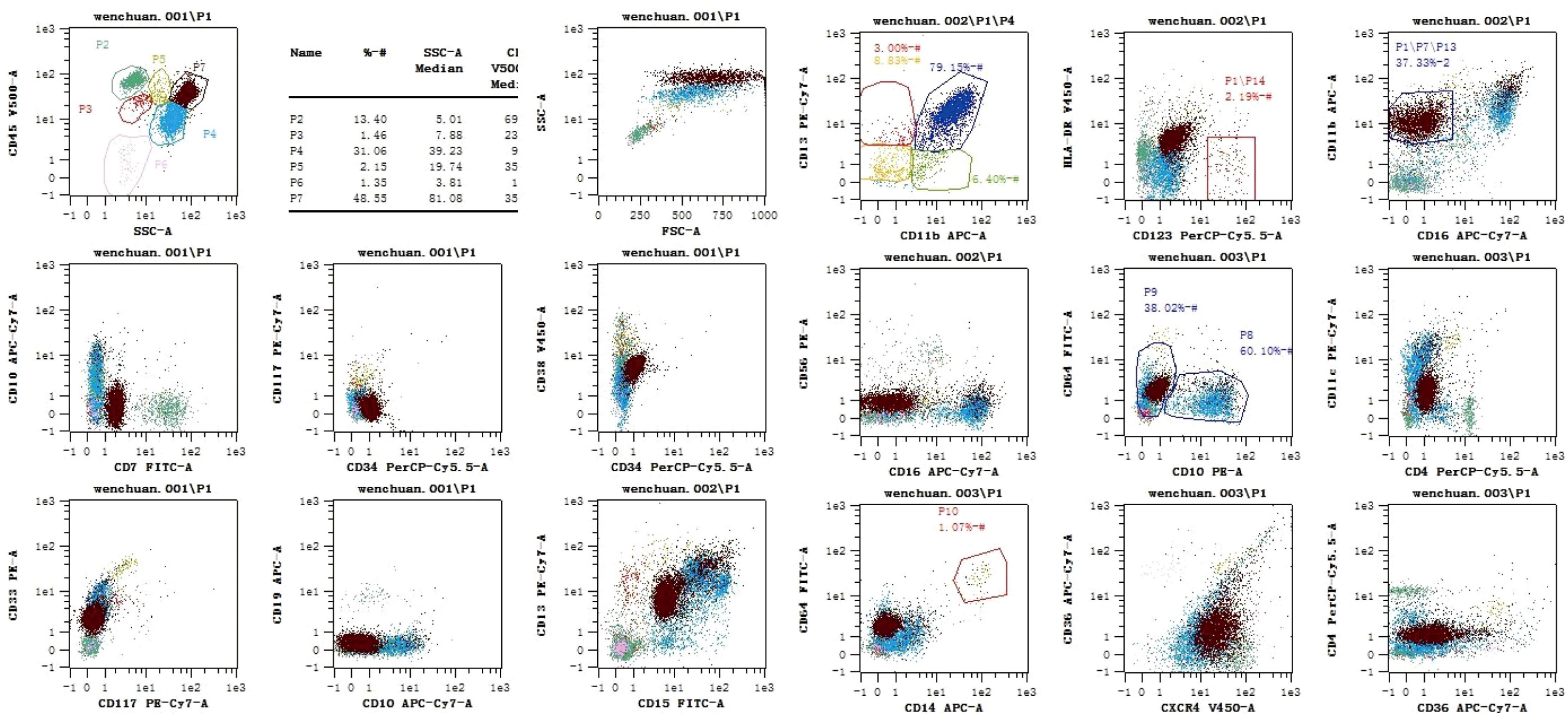
Immunophenotyping. Lymphocytes (P2) account for 13.40% of all cells (normal proportion), and consist mainly of mature T lymphocytes. Myeloid cells (P4) account for 31.60% of all cells (decreased proportion). A normal proportion of CD10+ mature granulocytes is seen, with decreased expression of CD15. Monocytes account for 1.07% of all cells, and their phenotype is not significantly abnormal. Nucleated red blood cells account for 1.35% of all cells (decreased proportion). No CD34+ CD117+ immature myeloid cells are found. Basophils account for 2.19% of all cells, and eosinophils account for 37.33% of all cells (significantly increased proportion).

**Figure 4:**
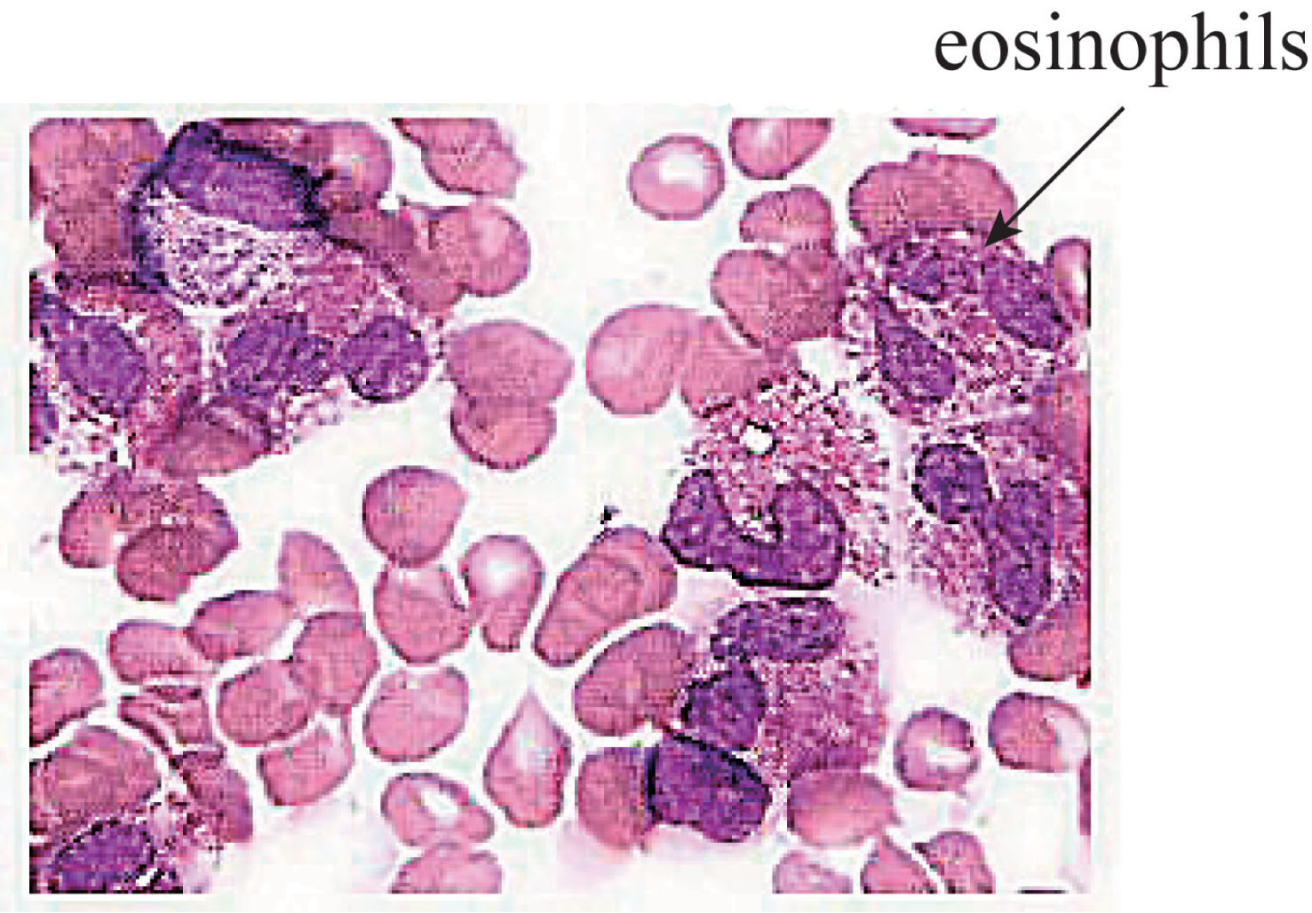
Morphological description and hemogram of bone marrow smear: bone marrow hyperplasia is slightly decreased; the proportion of eosinophils is increased; and there are few platelets. Morphological description (1): Myeloproliferative grade III–IV. Granulocytes account for 87.00% of the cells, while erythroid cells account for 2.50% of all cells. Granulocytes: red = 34.80:1. (2) In the granulocyte lineage, cells can be observed below the promyelocyte stage, with a notable increase in the proportion of eosinophils. (3) Erythroid lineage: a small number of middle and late immature erythrocytes can be seen, and mature erythrocytes show varying morphology and size. (4) The proportion of lymphocytes is normal. (5) Monocytes and plasma cells can be seen. (6) There are no macrophages and few platelets in the whole film. Hemogram: (1) There are many white blood cells, and the proportion of eosinophils is significantly increased. (2) Mature red blood cells show varying shapes and sizes. (3) Few platelets are seen.

Brain MRI performed on Day 2 revealed multiple abnormal signals in the left cerebellar hemisphere and right frontal and temporal lobes, indicating an acute-subacute infarction (**Figures 2A–D**). Comprehensive transthoracic echocardiography (see **Supplementary Table 2**) showed no structural abnormalities, intracardiac thrombi, or valvular dysfunction, definitively excluding a cardioembolic source. Cervical vascular ultrasonography found no clear abnormalities in the carotid arteries on both sides. Transcranial Doppler (TCD) showed a narrowed spectrum in the M1 segment of the right middle cerebral artery (**Supplementary Figure 3**). Intracranial magnetic resonance angiography (MRA) indicated vascular thinning from the right internal carotid to the middle cerebral artery, with weaker blood-flow signals compared to the opposite side and reduced flow in distal branches (**Figure 2E**). We specifically aimed to investigate the presence of eosinophilic infiltrates, which is crucial for the diagnosis and understanding of the patient’s condition. In order to observe whether the proportion of eosinophils in the bone marrow was increased, we performed a bone marrow biopsy. The results revealed decreased cell proliferation, high eosinophil levels, and low platelet counts. Flow cytometric immunophenotyping confirmed a significant expansion of eosinophils without evidence of an immature myeloid population (see **Figure 3** for full data). Morphological details are shown in **Figure 4**, and quantitative data in **Supplementary Table 3**. Bone marrow biopsy images revealed histopathologic characteristics of myeloid sarcoma. The pathologic examination indicated active hematopoietic tissue proliferation (**Supplementary Figure 2**). The *F/P* fusion gene was detected using a genetic assay on Day 11. This fusion gene was generated by a cryptic deletion at 4q12, linking exon 7 of FIP1L1 to exon 12 of PDGFRA, and resulted in a constitutively active PDGFRA receptor tyrosine kinase that promotes eosinophil production. Breakpoint confirmation was obtained using an in-house reverse transcription polymerase chain reaction (RT-PCR) assay established by the Peking University Institute of Hematology, based on previously published primers and probes. The amplified product was subsequently confirmed by Sanger sequencing, which verified the in-frame fusion transcript. The same testing also documented high WT1 expression and normal PRAME levels (**Supplementary Table 4**).

The patient was diagnosed with acute cerebral infarction and *F/P*+ MN-eo after MRI, blood tests, a bone marrow smear, and a *FIP1L1-PDGFRA* fusion gene test. Beginning on Day 15, he has been taking 0.1 g/day of oral imatinib, which has significantly improved his symptoms, blood test results, and enzyme levels (**Supplementary Figures 1B, C**). After 9 years of follow-up, his limb muscle strength is normal, and he has no other discomfort or adverse genetic effects. The FIP1L1-PDGFRA fusion transcript has become undetectable, with normalization and stabilization of eosinophil counts and percentages (**Supplementary Figure 1**). The patient is experiencing favorable health outcomes and has recently had a child.

## Discussion

Ischemic strokes linked to myeloid tumors with the *F/P* fusion gene are typically due to cardioembolic occlusion from mural thrombi caused by endomyocardial fibrosis, often affecting the watershed region. However, according to the literature, the possible pathogenesis of eosinophilia leading to cerebral infarction is as follows (4–6): (a) Eosinophilia may lead to cerebral infarction through increased blood hypercoagulability, as eosinophils release proteins and substances that raise blood viscosity. Major basic protein damages vascular endothelial cells, exposing tissue factor and activating factor XII, which triggers the extrinsic coagulation pathway. This inhibits thrombomodulin, reduces the inactivation of coagulation factor V, and leads to blood hypercoagulability. Eosinophilic cationic protein further inhibits thrombomodulin’s anticoagulant activity, enhancing the procoagulant effect. (b) Cardiogenic embolism: Eosinophil toxins damage heart cell membranes and mitochondrial enzymes, causing myocardial remodeling and fibrosis. Eosinophil-produced peroxidase, free radicals, trypsin, and collagenase harm the heart lining and muscle, leading to thrombosis, shedding, and cardiogenic stroke. (c) Local thrombotic dysplasia involves eosinophils expressing CD40 ligand and von Willebrand factor, and releasing collagen and tissue factor, all of which contribute to thrombosis. Eosinophils directly damage endothelial cells, exposing collagen and causing injury and thrombosis. They also induce inflammatory and tissue factors, leading to vascular endothelial injury, inflammation, and vascular wall damage. (d) Hypoperfusion: Eosinophils can damage microcirculation and cause microembolisms, resulting in poor blood flow to small cerebral vessels. (e) Platelet activation: Eosinophils quickly gather at arterial thrombus sites, interact with platelets, and form extracellular traps. These traps activate and aggregate platelets via eosinophil cationic protein and major basic protein. Eosinophilia is a multisystem disorder characterized by persistent clonal proliferation of eosinophils, leading to high eosinophil levels in blood, bone marrow, and tissues. The 2022 WHO criteria diagnose eosinophilia with a peripheral blood eosinophil count of ≥1.5 × 10^9^/L for at least 4 weeks (7). A myeloid neoplasm with the *FIP1L1–PDGFRα* gene rearrangement is a rare cause of HE; this condition is characterized by peripheral eosinophilia, defined as an elevation of the eosinophil count above 1.5 × 10^3^/μL, and the presence of the *FIP1L1–PDGFRα* fusion gene, which is identified using fluorescent *in situ* hybridization.

Our patient experienced multiple strokes in the left cerebellar hemisphere and right frontotemporal lobe, despite lacking common cerebrovascular risk factors. No thrombus was found via echocardiography, ruling out a cardioembolic source. The strokes might be due to early cardiac inflammation or undetected wall thrombi in areas like the left atrial appendage, which are difficult to visualize with transthoracic echocardiography. Drug therapies for myeloid tumors with eosinophilia and the FIP1L1-PDGFRA fusion gene include chemicals, glucocorticoids, interferons, and tyrosine kinase inhibitors, with the latter targeting fusion gene therapy to help patients achieve biological remission (8). Imatinib mesylate (Gleevec) effectively treats the disease, with sensitivity varying by gene type. The FIP1L1-PDGFRA fusion gene is highly responsive, typically achieving remission with 50–200 mg/day, usually 100 mg/day. Imatinib inhibits FIP1L1-PDGFRA but doesn’t completely eliminate its abnormal expression, leading to recurrences in F/P+ MN-eo patients due to the α fusion gene and treatment interruptions. The optimal dose and maintenance effect of imatinib remain uncertain, requiring further study (9). Aspirin antiplatelet therapy has been used in the context of stroke in undetermined etiology. Literature suggests a 400 mg/day dose for treating PDGFRB CEL with positive gene rearrangement. In contrast, imatinib treatment is ineffective for CEL with *PDGFR1* gene rearrangement, and patients with this condition can receive chemotherapy or undergo hematopoietic stem cell transplantation (10).

A recent large cohort study by Rohmer et al. provides important epidemiological context, reporting that central nervous system (CNS) involvement, predominantly ischemic stroke, occurs in approximately 9% of patients with F/P+ neoplasms (11). Among these, multi-territorial strokes are common, underscoring the systemic pro-thrombotic state driven by eosinophilia. This report rightly contextualizes our case and indicates that stroke, while rare, is a recognized presenting feature of this disease. However, our case presents several distinctive and under-reported features that expand the clinical spectrum: First, the stroke in our patient was the initial and sole presenting symptom leading to the diagnosis, without concurrent signs of cardiac involvement (such as the left ventricular thrombus noted in some patients in the cited series) at presentation. Second, neuroimaging revealed a focal stenosis of the right middle cerebral artery, suggesting a localized eosinophil-mediated vasculopathic injury rather than a cardioembolic mechanism, which is less commonly documented. Most notably, this is, to our knowledge, the first report of a non-leukemic myeloid sarcoma phenotype presenting as acute cerebral infarction in this disease context. Finally, the patient achieved a sustained molecular and clinical remission for over 9 years with low-dose imatinib monotherapy alone, without requiring any anticoagulant or antiplatelet agents, demonstrating an exemplary long-term management outcome.

This case report describes the first documented instance of a non-leukemic myeloid sarcoma associated with FIP1L1-PDGFRA rearrangement presenting as acute cerebral infarction, a unique phenotype expanding the clinicopathological spectrum of F/P+ MN-eo. Critically, the patient achieved sustained cytogenetic and molecular remission for over 9 years using low-dose imatinib monotherapy (100 mg/day) alone, representing one of the longest and most comprehensively documented cases of sustained remission using low-dose imatinib monotherapy without adjunctive therapies, thereby reinforcing its efficacy as a definitive long-term strategy for this condition (12). This outcome is consistent with the excellent long-term responses to imatinib reported in larger cohorts (11, 13). Unlike prior reports attributing strokes to cardioembolism (14–16), multimodal imaging revealed right middle cerebral artery stenosis without cardiac thrombi, suggesting eosinophil-mediated vascular injury through hypercoagulability and endothelial damage, a mechanism supported by multimodal evidence. While histopathologic confirmation of vascular eosinophilic infiltration was not obtained due to the absence of brain biopsy, the collective evidence—including (a) significant eosinophilia (peak 51.4%), (b) right middle cerebral artery stenosis on MRA without atherosclerotic risk factors, (c) elevated d-dimer (752 ng/mL) and lactate dehydrogenase (419 U/L) indicating hypercoagulability and tissue damage, and (d) rapid resolution of stenosis after imatinib treatment—supports this mechanistic hypothesis. Prior studies have documented eosinophil-induced endothelial injury in cerebral vessels (4). These findings collectively validate early F/P fusion testing in unexplained stroke with eosinophilia, demonstrate the sufficiency of targeted monotherapy for durable remission, and redefine myeloid sarcoma as a treatable component of this neoplasm.

In summary, cerebrovascular disease is seldom the first symptom of HE. Leukemia patients typically develop bleeding due to coagulation problems and low platelet counts, making thrombosis rare in this population. Our patient presented with acute cerebral infarction, and had no usual cerebrovascular risk factors, but exhibited elevated eosinophil levels. In this case, a prompt diagnosis necessitated the identification of eosinophilia, a bone marrow examination, and the early detection of the FIP1L1-PDGFRA rearrangement. Consequently, the patient was administered imatinib at a daily dosage of 100 mg. Following three weeks of treatment, both eosinophilia and myeloid sarcoma were completely resolved, and a subsequent evaluation at three months demonstrated bone marrow cytogenetic remission. This report details a unique presentation of F/P+ MN-eo, characterized by the triad of acute cerebral infarction as the initial manifestation, associated myeloid sarcoma, and middle cerebral artery stenosis in the absence of cardioembolism. Although myeloid sarcoma is typically characterized by aggressive clinical behavior, the presence of a PDGFRA rearrangement in this particular case resulted in a high sensitivity to imatinib treatment, leading to a favorable clinical outcome. Our study underscores the importance of fusion gene detection for effective diagnosis and treatment.

## Data Availability

The raw data supporting the conclusions of this article will be made available by the authors, without undue reservation.

## Supplementary material

Supplementary Figure 1 Longitudinal monitoring of molecular and hematological parameters over 9 years of imatinib therapy. The patient has been taking 0.1 g/day of oral imatinib, which has significantly improved his symptoms, blood test results, and enzyme levels. **(A)** Trends in FIP1L1-PDGFRA fusion transcript levels, expressed as a percentage of the *ABL* reference gene. **(B)** Percentage of eosinophils in peripheral blood over time. **(C)** Absolute eosinophil count (×10^9^/L).

Supplementary Figure 2 Active proliferation of hematopoietic tissue.

Supplementary Figure 3 **(A)** Transcranial Doppler (TCD)-1 (speed: cm/s): The right middle cerebral artery has moderate stenosis (arrows). **(B)** TCD-2 (speed: cm/s): No obvious abnormality is found in the blood flow spectrum of the left and right cervical vessels.

Supplementary Table 1 Laboratory findings of the patient.

Supplementary Table 2 Echocardiogram index. CDFI, color Doppler flow imaging.

Supplementary Table 3 Bone marrow smear (posterior superior, Wright’s staining), The average value represents the mean value of the counts, while the SD (standard deviation) indicates the variability of these counts.

Supplementary Table 4 Fusion gene RT-PCR data. In addition: Detection of CALR gene by qualitative PCR. Mutation of exon 9 gene. The result is negative.
