# Supplementary figures and images for: Acute hemiplegia as initial presentation in FIP1L1-PDGFRA-rearranged myeloid neoplasm with eosinophilia: a case report
Source: Front Oncol. 2026 Feb 10;16:1628690. doi: 10.3389/fonc.2026.1628690 (PMC12929143; doi:10.3389/fonc.2026.1628690)

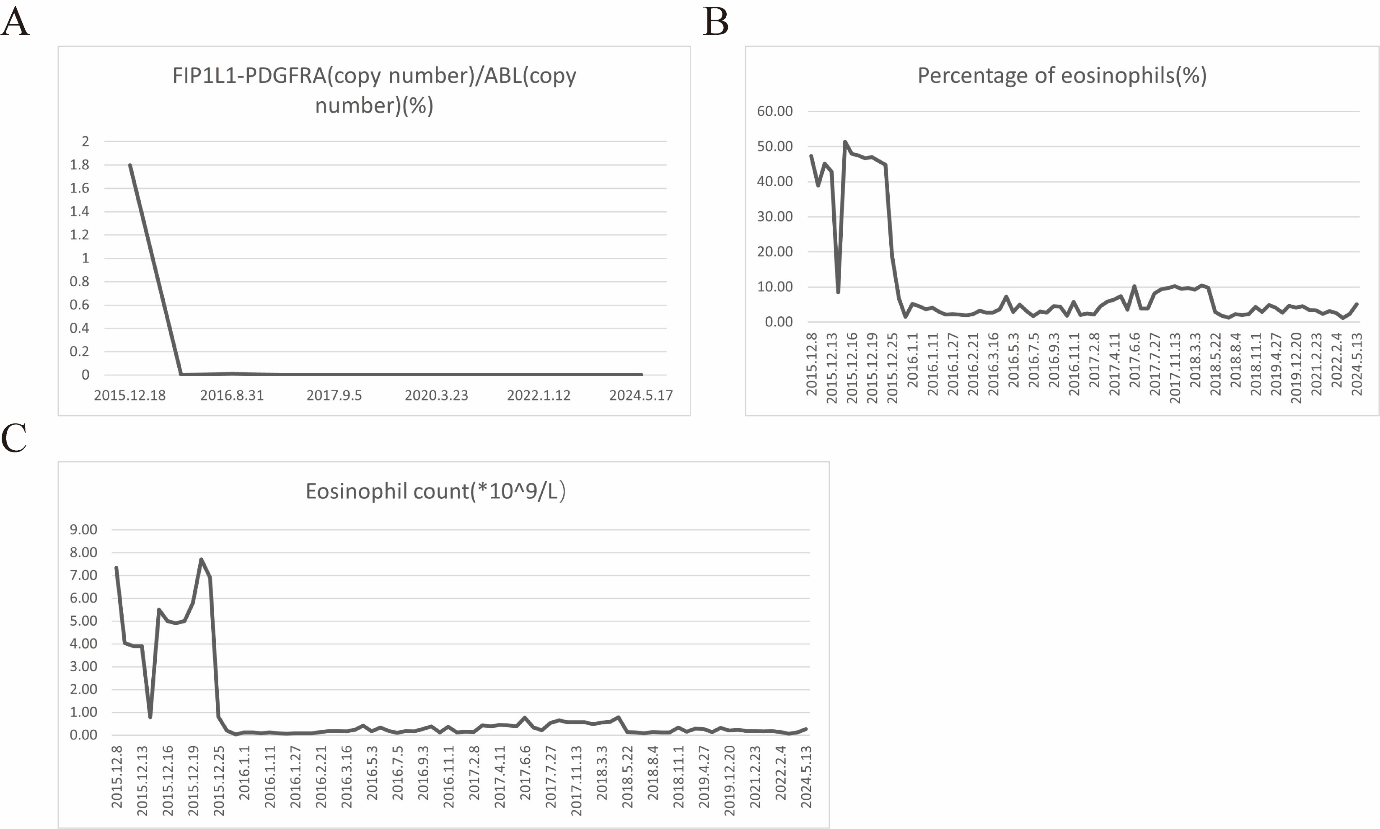

Supplement: Supplementary Figure 1 — Longitudinal monitoring of molecular and hematological parameters over 9 years of imatinib therapy. The patient has been taking 0.1 g/day of oral imatinib, which has significantly improved his symptoms, blood test results, and enzyme levels. (A) Trends in FIP1L1-PDGFRA fusion transcript levels, expressed as a percentage of the ABL reference gene. (B) Percentage of eosinophils in peripheral blood over time. (C) Absolute eosinophil count (×109/L). [file Image1.tif]

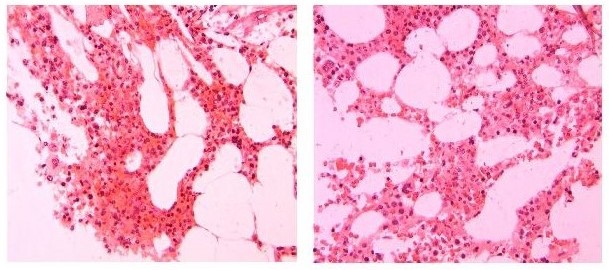

Supplement: Supplementary Figure 2 — Active proliferation of hematopoietic tissue. [file Image2.jpeg]

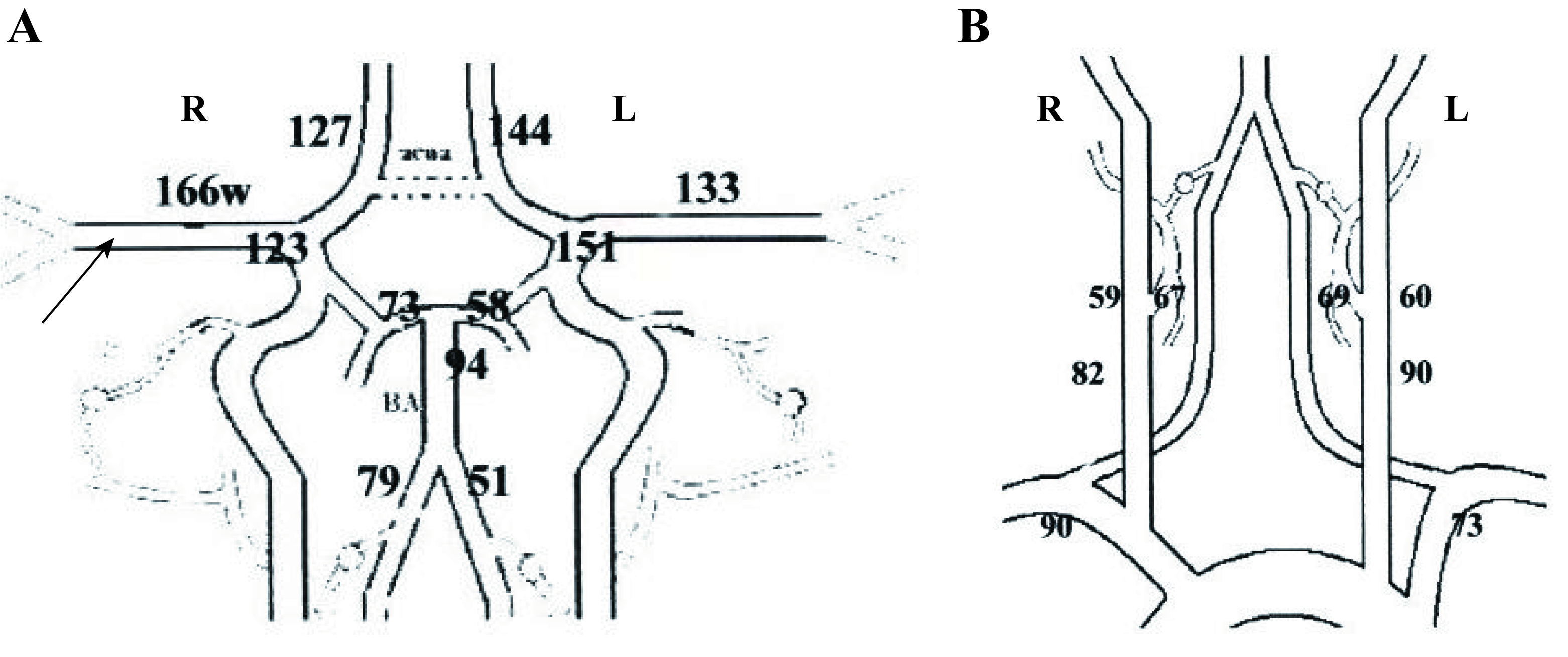

Supplement: Supplementary Figure 3 — (A) Transcranial Doppler (TCD)-1 (speed: cm/s): The right middle cerebral artery has moderate stenosis (arrows). (B) TCD-2 (speed: cm/s): No obvious abnormality is found in the blood flow spectrum of the left and right cervical vessels. [file Image3.tif]
