# Supplementary material for: Acute hemiplegia as initial presentation in FIP1L1-PDGFRA-rearranged myeloid neoplasm with eosinophilia: a case report
Source: Front Oncol. 2026 Feb 10;16:1628690. doi: 10.3389/fonc.2026.1628690 (PMC12929143; doi:10.3389/fonc.2026.1628690)
Supplement: Supplementary Table 1 — Laboratory findings of the patient. [file Table1.pdf]

Supplemental Table 1. Laboratory findings of the patient.

**Date**	**Day -195**	**Day 3**	**Day 5**	**Day 8**	**Day 12**	**Day 16**	**Day 22**	**Day 29**	**Day 35**
White blood cell (*10^9/L)	6.45	10.36	8.7	10.7	12.3	15.3	2.61	2.44	2.94
Hemoglobin (g/L)	140	96	103	98	99	97	104	106	115
Platelet (*10^{12}/L)	203	80	68	58	72	94	205	218	174
Eosinophil count (*10^9/L)	1.55	4.03	3.90	5.50	5.80	6.90	0.04	0.11	0.12
Percentage of eosinophils (%)	24	38.9	45.2	51.4	47	44.8	1.5	4.5	4.1
