# Supplementary material for: Acute hemiplegia as initial presentation in FIP1L1-PDGFRA-rearranged myeloid neoplasm with eosinophilia: a case report
Source: Front Oncol. 2026 Feb 10;16:1628690. doi: 10.3389/fonc.2026.1628690 (PMC12929143; doi:10.3389/fonc.2026.1628690)
Supplement: Supplementary Table 2 — Echocardiogram index. CDFI, color Doppler flow imaging. [file Table2.pdf]

Supplemental Table 2. Echocardiogram index

<table>
<tr><td colspan="4">Echocardiogram</td></tr>
<tr><td colspan="4">M-type/2D measurement and calculation</td></tr>
<tr><td>Main pulmonary artery diameter: 2.2 cm</td><td colspan="2">End-diastolic thickness of interventricular septum: 0.76 cm</td><td>Ejection fraction (Teich): 70.3%</td></tr>
<tr><td>Diameter of aorta: 3.0 cm</td><td colspan="2">Left ventricular end-diastolic diameter: 5.0 cm</td><td>End-diastolic volume (Teich): 116.2 mL</td></tr>
<tr><td>Diameter of ascending aorta: 2.8 cm</td><td colspan="2">Left ventricular end-systolic diameter: 3.0 cm</td><td>End-systolic volume: 34.6 mL</td></tr>
<tr><td>Anterior-posterior diameter on the left side: 3.2 cm</td><td colspan="2">Left ventricular posterior wall thickness: 0.94 cm</td><td></td></tr>
<tr><td></td><td colspan="2">Left ventricular mass: 145.1 grams.</td><td></td></tr>
<tr><td colspan="4">Doppler measurement and calculation</td></tr>
<tr><td colspan="2">Maximum velocity above aortic valve: 121.9 cm</td><td colspan="2">Mitral valve E-peak velocity: 87.5 cm/s</td></tr>
<tr><td colspan="2">Aortic peak pressure gradient: 5.9 mm Hg</td><td colspan="2">Mitral valve A-peak velocity: 60.6 cm/s</td></tr>
<tr><td colspan="2"></td><td colspan="2">Mitral valve E/A value: 1.4</td></tr>
<tr><td colspan="4">Great vessels: Diameter of aorta is normal. Main pulmonary artery diameter is normal.</td></tr>
<tr><td colspan="4">Cordis: Left atrial diameter is normal. Right atrial diameter is normal.</td></tr>
<tr><td colspan="4">Left ventricle: Left ventricular diameter is normal. Left ventricular thickness is normal. The systolic function of the left ventricle has no obvious abnormality when resting. A false tendon was found in the apical area of the left ventricle.</td></tr>
<tr><td colspan="4">Right ventricle: Right ventricular diameter is normal.</td></tr>
<tr><td colspan="4">Bicuspid valve: No obvious abnormalities were found in the bicuspid structure. CDFI: No backstreaming was found in the bicuspid valve.</td></tr>
<tr><td colspan="4">Tricuspid valve. No obvious abnormalities were found in the tricuspid structure. CDFI: No backstreaming was found in the tricuspid valve.</td></tr>
<tr><td colspan="4">Aortic valve: No obvious abnormalities were found in the aortic structure. CDFI: No backstreaming was found in the aortic valve.</td></tr>
<tr><td colspan="4">Pulmonary valve: No obvious abnormalities were found in the pulmonary structure. CDFI: No backstreaming was found in the pulmonary valve.</td></tr>
<tr><td colspan="4">Pericardium: No thickening of the pericardium is observed. No anechoic area or other abnormal echoes were found in the pericardial cavity.</td></tr>
<tr><td colspan="4">Conclusion: No obvious abnormalities were found in the cardiac structure and blood flow.</td></tr>
</table>

CDFI, color Doppler flow imaging
