# Supplementary material for: Acute hemiplegia as initial presentation in FIP1L1-PDGFRA-rearranged myeloid neoplasm with eosinophilia: a case report
Source: Front Oncol. 2026 Feb 10;16:1628690. doi: 10.3389/fonc.2026.1628690 (PMC12929143; doi:10.3389/fonc.2026.1628690)
Supplement: Supplementary Table 3 — Bone marrow smear (posterior superior, Wright’s staining), The average value represents the mean value of the counts, while the SD (standard deviation) indicates the variability of these counts. [file Table3.pdf]

Supplemental Table 3. Bone marrow smear (posterior superior, Wright's staining)

Cell			Blood (%)	Bone marrow smears		
				Average value	SD	%
Primitive blood cells				0.08	0.01	
Granulocyte series	Myeloblast			0.64	0.33	
	Promyelocytic			1.57	0.6	0.5
	Neutrophil	Myelocyte		6.49	2.04	9.5
		Metamyelocyte		7.90	1.07	3.0
		Stab form granulocyte	6.0	23.72	3.5	5.0
		Lobular granulocyte	12.0	9.44	2.92	16.0
	Acidophilia	Myelocyte		0.38	0.23	
		Metamyelocyte		0.49	0.32	
		Stab form granulocyte	5.0	1.25	0.61	
		Lobular granulocyte	68.0	0.05	0.61	51.5
	Basophilic	Myelocyte		0.02	0.05	
		Metamyelocyte		0.06	0.07	
		Stab form granulocyte		0.1	0.09	
		Lobular granulocyte	1.0	0.03	0.05	1.5
Erythrocytic series	Myeloblast			0.57	0.3	
	Promyelocytic			0.92	0.41	
	Intermediate			7.41	1.91	1.5
	Metamyelocyte			10.75	2.36	1.0
	Promyelocyte					
	Polychromatic megaloblast					
	Orthochromatic megaloblast					
Immune cell series	Myeloblast			0.06	0.09	
	Immature			0.47	0.64	
	Mature		5.0	22.78	7.04	0.5
Mononuclear series	Myeloblast			0.01	0.04	
	Immature			0.14	0.19	
	Mature		3.0	3	0.05	0.5
Megakaryocyte series	Myeloblast					
	Immature					
	Platelet-producing					
	Granular					
	Naked					

Plasma cell	Myeloblast		0.004	0.02	
	Immature		0.104	0.16	
	Mature		0.71	0.42	0.5
Other	Reticulum		0.05	0.09	
	Megakaryocyte				
	Phagocyte				
	Tissue basophils				
	Adipocyte				
	Unknown		0.03	0.09	
Granulocyte series: nucleated red blood cell		92.0	2.76	0.87	34.50:1
Total number of cells		100	cell		200

The average value represents the mean value of the counts, while the SD (standard deviation) indicates the variability of these counts.
