# Supplementary material for: Acute hemiplegia as initial presentation in FIP1L1-PDGFRA-rearranged myeloid neoplasm with eosinophilia: a case report
Source: Front Oncol. 2026 Feb 10;16:1628690. doi: 10.3389/fonc.2026.1628690 (PMC12929143; doi:10.3389/fonc.2026.1628690)
Supplement: Supplementary Table 4 — Fusion gene RT-PCR data. In addition: Detection of CALR gene by qualitative PCR. Mutation of exon 9 gene. The result is negative. [file Table4.pdf]

Supplemental Table 4. Fusion gene RT-PCR data

Reference gene ABL (copy number) = 32680	
The results of real-time quantitative PCR detection of gene expression are as follows:	
BCR-ABL (P-190) = 0	BCR-ABL (P210) = 0
BCR-ABL (P230) = 0	BCR-ABL (variant) = 0
FIP1L1-PDGFRA = 579/32680 = 1.8%	TEL-PDGFRB = 0
CBFB-MYH11 = 0	WT1/ABL-627/32680 = 1.9% (normal value < 0.6%)
PRAME/ABL = 158/32680 = 0.48% (normal value < 0.3%)	
Brief summary<br>Good internal reference of the specimen<br>FIP1L1-PDGFRA fusion gene: abnormal high expression of WT1, PRAME expression is generally normal<br>Suggest following up on FIP1L1-PDGFRA mRNA levels to monitor residual diseases	

In addition: Detection of CALR gene by qualitative PCR. Mutation of exon 9 gene. The result is negative.
